# Performance indicators for organ donation and transplantation programmes in Europe: modified Delphi consensus study

**DOI:** 10.1093/bjs/znaf293

**Published:** 2026-01-22

**Authors:** Simon Streit, George Wharton, Jasmine Mah, Robin van Kessel, Apostolos Prionas, Charlotte Johnston-Webber, John Boletis, Beatriz Domínguez-Gil, Anna Forsberg, Ana França, Dale Gardiner, Patrick Jeurissen, Irene Papanicolas, Oliver Pearcey, Allan Rasmussen, Jacopo Romagnoli, Elias Mossialos, Vassilios Papalois

**Affiliations:** Institut für Neuropathologie, Charité Universitätsmedizin, Berlin, Germany; Department of Health Policy, London School of Economics and Political Science, London, UK; Division of Geriatric Medicine, Dalhousie University, Halifax, Nova Scotia, Canada; Department of Health Policy, London School of Economics and Political Science, London, UK; Department of Surgery, Imperial College, London, UK; Department of Health Policy, London School of Economics and Political Science, London, UK; Transplantation Unit, Laiko Hospital, Athens, Greece; Organización Nacional de Trasplantes, Madrid, Spain; Department of Health Sciences, Lund University, Lund, Sweden; Instituto Português do Sangue e da Transplantação, Lisbon, Portugal; NHS Blood and Transplant, Bristol, UK; Radboud Institute for Health Sciences, IQ Healthcare Scientific Institute for Quality of Healthcare, Nijmegen, The Netherlands; Department of Health Policy, London School of Economics and Political Science, London, UK; West London Kidney Patients’ Association, London, UK; Department of Surgical Gastroenterology and Transplantation, Rigshospitalet, University of Copenhagen, Copenhagen, Denmark; Dipartimento di Scienze Mediche e Chirurgiche, Fondazione Policlinico Universitario A. Gemelli IRCCS, U.O.S. Trapianti di Rene, Roma, Italy; Department of Health Policy, London School of Economics and Political Science, London, UK; Department of Surgery, Imperial College, London, UK

## Abstract

**Background:**

Health system performance assessment helps identify areas for improvement and guides policy initiatives. Although well-validated indicators exist for measuring organ donation and transplantation performance at the facility level, consensus on indicators for assessing national programmes is lacking. The aim of this study was to develop a comprehensive scorecard for evaluating national organ donation and transplantation programmes.

**Methods:**

A three-step approach was used. First, a targeted literature review identified potential indicators from regulatory documents, national transplant organization reports, and databases. Second, indicators were mapped to an established transplant system framework and refined through preliminary expert consultations. Third, a modified Delphi consensus process validated the indicators. The Delphi panel comprised international experts in health policy, organ donation, transplantation, and patient representation. Participants rated 168 indicators using a five-point Likert scale across two rounds (24 experts completed round 1 and 22 experts completed round 2). Consensus for inclusion required 80% agreement.

**Results:**

Of 168 indicators evaluated, 103 achieved consensus for inclusion. After consolidation of organ-specific indicators, the final set contained 84 indicators across seven domains: monitoring and reporting (8 indicators), prevention and need (9 indicators), waiting lists (11 indicators), consent (4 indicators), donation (28 indicators), transplantation (14 indicators), and follow-up (10 indicators). The indicator set incorporates established metrics such as waiting list statistics, donation rates, and complication rates alongside novel system-level indicators addressing structural factors, patient-centredness, and equity in care delivery.

**Conclusion:**

This validated indicator set provides a standardized tool for assessing and comparing transplant system performance across European countries, supporting performance benchmarking and evidence-informed policy development.

## Teaser

This study developed a comprehensive scorecard of 84 validated performance indicators across seven domains to evaluate national organ donation and transplantation programmes. Using a modified Delphi consensus process with 30 international experts, researchers identified indicators that address both established metrics (such as donation rates and waiting list statistics) and novel system-level factors, including equity and patient-centredness. The validated indicator set provides a standardized tool for benchmarking transplant system performance across European countries and supporting evidence-informed policy development.

## Introduction

Health system performance assessment serves multiple important functions in the governance and management of health systems. Robust performance measurement frameworks are vital for accountability and transparency. They enable stakeholders to track progress over time, identify areas requiring attention, prioritize resource allocation, evaluate policy interventions, and compare performance across settings^1–8^. These functions support both internal quality improvement and cross-system learning.

For organ donation and transplantation, national programmes vary substantially between countries within and across global regions^3,9,10^. In Europe, countries such as Spain have established comprehensive organ donation frameworks and rigorous standards, enabling thousands of transplantation procedures annually. In contrast, patients in many other European countries continue to face severely limited access to these life-saving interventions^9,11–14^.

These disparities cannot be explained solely by healthcare resources. For instance, countries like Croatia and Portugal have achieved notably high donation rates despite facing resource constraints^15,16^. This highlights the potential for comparing transplant systems to identify opportunities for cross-country learning^10^.

Accordingly, various efforts have been made to distil and apply crucial components of transplant system reform across both European and non-European countries^10,17–20^. For instance, a team of researchers (including authors of the present study) used case studies of successful transplant systems to inform policy recommendations to strengthen the Greek system^13–16,21,22^. Through these reviews of different organ donation and transplantation programmes, the authors recognized the need for quantitative indicators to assess transplant system status. A national scorecard offering a concise overview of system performance would complement qualitative evaluations and enable continuous performance monitoring.

To date, indicator development in transplantation has focused on the performance of individual centres, yielding a comprehensive list of validated indicators reflecting clinical pathways^18,20,23,24^ . Key reports in this field have highlighted the potential for broadening this scope, including the ‘need to perform international comparisons, […] [and] agree on a minimum common set of indicators to be constructed and compared in the future’^20^. Yet at a health system or country level, performance indicators remain less well developed.

The Global Observatory on Organ Donation and Transplantation (GODT) provides a valuable resource by compiling worldwide donation and transplantation rates^9,25^. Although these rates serve as intuitive indicators of transplant system effectiveness, they do not capture the full complexity of transplant systems. Specifically, these rates do not reflect patient experience or post-transplantation quality of life. They also do not consider social inequalities in access to transplantation or efforts to reduce the need for transplants. Other approaches to capturing transplant system performance have overcome these limitations, but have focused solely on kidney transplantation or on refinement of existing donation metrics alone^26,27^.

The aim of this study was to develop a comprehensive set of organ donation and transplantation performance indicators to support performance assessment, international comparison, and evidence-informed policy development.

## Methods

### Ethics

This study involved literature review and expert consultation through a modified Delphi process. All participants provided informed consent before participation. Responses were anonymized via Welphi, a secure online platform. No personal data were collected and participants could withdraw at any time. As this study involved professional experts providing opinions on health system indicators rather than research on human subjects, formal ethics committee approval was not sought. The study was conducted in accordance with good research practice guidelines.

### Study design

A three-step approach was employed to develop a comprehensive set of indicators. First, a targeted literature review was undertaken to identify indicators of organ donation and transplantation system performance. Second, the indicators were mapped to an established framework for national transplantation systems and experts were consulted to establish face validity, refine existing indicators, and identify missing indicators. Third, an adapted Delphi technique was used to prioritize the indicators based on expert consensus. This study is reported in accordance with the STROBE guidelines for cross-sectional studies (see *Table S2* for checklist).

### Literature review

A targeted review of grey literature focused on international resources related to organ donation and transplantation. These resources included the GODT, the comprehensive set of quality indicators outlined in the Organ Donation European Quality System (ODEQUS) project, the Council of Europe Guide to the Quality and Safety of Organs for Transplantation, and the European Renal Association Registry^9,18,23,28,29^.

The review also included websites and national reports from national transplant organizations (NTOs) in ten countries: Australia, Canada, Croatia, France, Germany, Italy, Portugal, Spain, the UK, and the USA. These countries were selected to include a diverse set of health systems relevant to the European context, based on accessibility of national reports to the research team.

Additionally, the review included general health system indicators routinely collected by the European Commission and the Organisation for Economic Co-operation and Development (OECD), which were assessed for their relevance to organ donation and transplantation^28–32^.

### Indicator mapping and preliminary expert consultations

To assemble a comprehensive set of indicators, the aim was to cover different health system goals, as well as different aspects of care in organ donation and transplantation. For this purpose, the indicators were mapped to an established conceptual framework for organ donation and transplantation systems^33^. Following this framework, ‘improving health of patients with organ failure’ was defined as the key objective of a transplant system and indicators were categorized according to operational elements (‘prevention’, ‘donation’, ‘transplantation’, and ‘post-transplant follow-up’) and instrumental goals (‘responsiveness’, ‘efficiency’, and ‘equity’) defined in the framework^33^. This a priori mapping to an existing framework allowed identification of both well-represented areas and gaps across the donation-transplantation continuum.

An international expert panel was then consulted regarding the face validity of the indicators. Using purposive sampling, experts with diverse areas of expertise based in different geographical locations across Europe were consulted, concentrating on enlisting experts with whom the research team had prior collaborations. This included experts with clinical experience in organ donation and transplantation, as well as experts knowledgeable in managing national organ donation and transplantation programmes, health policy research, health system performance measurement, and patient representation.

Experts were provided with the indicator set from the review and asked to assess the importance of each indicator for measuring national transplant system performance on a scale from zero (least important) to ten (very important). This preliminary ranking exercise allowed subsequent open discussions and indicator refinements to focus on the most relevant indicators. Experts were also asked to suggest additional indicators they believed were missing from the list. After this step, the expert group was convened virtually to deliberate on top-ranked indicators by category, including refining indicator definitions, suggesting literature in areas where indicators were lacking, and recommending new indicators. The discussions were recorded and meeting minutes were circulated and approved by the experts. Based on these discussions, the experts were provided with a preliminary shortlist of top indicators and alternatives that had been discussed.

### Modified Delphi consensus process

To reach consensus on the final set of indicators and avoid exclusion of indicators not part of the preliminary discussions, a modified Delphi study was performed following established guidelines^34,35^. To ensure a robust consensus base, a wider group of experts were invited to participate, beyond those who took part in the preliminary consultations, ultimately including 30 international experts with backgrounds in transplantation clinical practice, health policy research, transplant system administration, and patient advocacy.

The consensus process was conducted between June and November 2024 using the Welphi platform, a specialized web application designed for Delphi studies^36^. Participants rated potential indicators on a five-point Likert scale: one (‘exclude’), two (‘maybe exclude’), three (‘uncertain’), four (‘maybe include’), and five (‘include’). Participants received comprehensive information, including indicator definitions and rationales for inclusion. To minimize order bias and context effects, indicators were presented in randomized order^37^. Participants could also provide qualitative comments on individual indicators.

Consensus for inclusion was defined as ≥80% of experts rating an indicator four or five (‘maybe include’ or ‘include’) and consensus for exclusion was defined as <50% of experts rating an indicator four or five. Statistical analysis was performed in R (R Foundation, Vienna, Austria), including both complete and incomplete responses to maximize data utilisation^34^.

After the first round, participant feedback was analysed, indicator definitions were refined, indicators deemed redundant were deleted, and new indicators were added based on expert suggestions. Indicators that achieved consensus for inclusion in the first round and were not edited or removed based on participant feedback were passed directly to the final shortlist. Indicators that were edited, newly added, or had moderate approval (50–79% of experts rating them 4 or 5) were carried forward to the second round of the survey. After completing the second round, additional indicators that achieved consensus were added to the final shortlist.

To further stratify indicators, priority was assigned based on a combination of median ratings, interquartile ranges, and the percentage of experts rating an indicator four or five. Priority was measured in four categories: very high priority (median rating = 5 and interquartile range = 0), high priority (indicators rated 4 or 5 by ≥80% of experts), moderate priority (those rated 4 or 5 by 50–79% of experts), and low priority (those rated 4 or 5 by <50% of experts).

In a final consolidation step, the indicator set was streamlined by consolidating organ-specific indicators that measured the same parameter across different organs (for example combining separate indicators for kidney, liver, and heart transplant volumes into a single comprehensive indicator for all organ types). This reduced redundancy in the primary indicator set while maintaining the ability to disaggregate data by organ type when needed for more targeted analysis.

## Results

Ten experts participated in the preliminary consultations (authors J.B., B.D.-G., A.Fo., A.Fr., D.G., P.J., I.P., O.P., A.R., and J.R.). After invitation, 30 experts agreed to participate in the modified Delphi process, including both external consultants and core research team members. Of these, 24 experts (80%) completed round one and 22 (73%) completed round two.

Participants included transplant surgeons, nephrologists, a geriatrician, a psychiatrist, a psychologist, and experts in public health, health policy, health services research, transplant coordination and system management, and patient advocacy. Approximately two-thirds of participants had clinical roles. Participants’ institutional affiliations were based across ten countries: Canada, Denmark, Germany, Greece, Italy, the Netherlands, Portugal, Spain, Sweden, and the UK.

### Consensus process and indicator selection

**Fig. 1:**
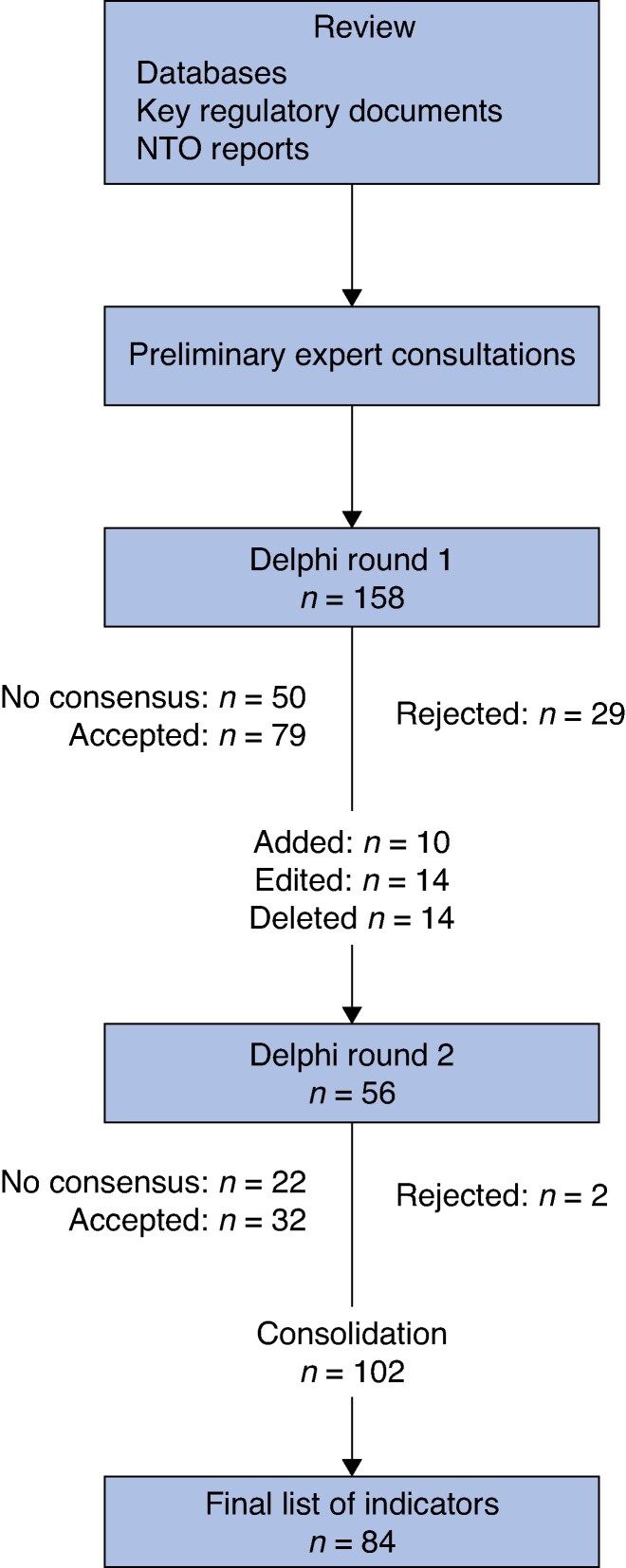
Modified Delphi consensus process NTO, national transplant organization.

*Figure 1* illustrates the consensus process. The initial literature review and preliminary expert consultations identified 158 indicators for evaluation in the first round of the survey. Based on expert comments during the first round, 10 indicators were added in the second round, 14 indicators were deleted, and 14 indicators were amended.

Of the 168 indicators evaluated across both rounds, 103 achieved consensus for inclusion (≥80% of experts rating them 4 or 5). After consolidation of organ-specific indicators that measured the same parameter across different organs, the final indicator set comprised 84 indicators distributed across seven domains: monitoring and reporting (8 indicators), prevention and need (9 indicators), waiting lists (11 indicators), consent (4 indicators), donation (28 indicators), transplantation (14 indicators), and follow-up (10 indicators). The complete set of 84 indicators with their priority classifications is presented in *Table 1*.

**Table 1:** List of 84 validated indicators for transplant system analysis

Category	Indicator	Priority	Median	Interquartile range	Percentage of experts rating an indicator four or five	Source
Monitoring and reporting	Patient satisfaction with transplant care recorded and used in transplant system performance assessment	Very high priority	5	0	100	Expert panel
	National IT platform dedicated to organ donation and transplantation in place	Very high priority	5	0	90.91	Expert panel
	NTO runs a potential donor audit	High priority	5	1	86.36	Expert panel
	Donation and transplantation data reported to GODT	High priority	5	1	87.5	Expert panel
	Patient-centredness used as an indicator of transplant system performance	High priority	5	1	86.36	Expert panel
	Number of organs transferred to other countries as part of organ exchange programmes	High priority	5	1	90.91	Expert panel
	Performance indicators collected by the NTO are reported by age, ethnicity, SES, geography, and sex	High priority	5	1	90.91	Expert panel
	Matters of equity covered in routine reporting by NTO	High priority	4	1	83.33	Expert panel
Prevention and need	Incidence of patients accepted for renal replacement therapy (pmp)	High priority	5	1	90.91	ERA-EDTA
	National prevention programme for liver disease in place	High priority	5	1	81.82	Expert panel
	Number of patients with end-stage kidney disease assessed for kidney transplantation (pmp)	High priority	5	1	95.83	Expert panel
	Number of patients with end-stage renal failure (pmp)	High priority	5	1	87.5	European Commission
	Prevalence of patients on renal replacement therapy (unadjusted for age and sex)	High priority	5	1	90.91	ERA-EDTA
	Prevalence of patients on renal replacement therapy (adjusted for age and sex)	High priority	5	1	90.91	ERA-EDTA
	National prevention programme for chronic kidney disease in place	High priority	4.5	1	95.45	Expert panel
	Number of patients with diabetes with kidney failure (pmp)	High priority	4.5	1	86.36	Expert panel
	Number of patients with end-stage renal failure receiving conservative management (pmp)	High priority	4	1	81.82	Expert panel
Waiting lists	Median days between being added to the waiting list and receiving transplant, for patients who received a transplant	Very high priority	5	0	87.5	OPTN
	Median time awaiting first transplant (by organ)	Very high priority	5	0	96	Expert panel
	Total number of patients on waiting lists for organ transplantation per year (by organ, pmp)	Very high priority	5	0	91.67	DSO
	Total number of waiting list registrations per year (by organ, pmp)	Very high priority	5	0	95.83	DSO
	Waiting list is managed according to standardized criteria and international guidelines	Very high priority	5	0	86.36	Expert panel
	Median time from registration as a living donor to determination of suitability in days	High priority	5	1	86.36	OPTN
	Number of new registrations on the donor register (pmp)	High priority	5	1	84	AOTA
	Number of patients who died while registered on a waiting list per year (by organ, pmp)	High priority	5	1	83.33	DSO
	Median time awaiting second transplant (by organ)	High priority	4.5	1	83.33	Expert panel
	Median days between patient evaluation and determination of suitability for transplant	High priority	4	1	84	OPTN
	Number of patients re-entering waiting lists (pmp)	High priority	4	1	84	DSO
Consent	Consent to organ donation, relative to number of people asked	Very high priority	5	0	88	AOTA
	Family refusal rate	Very high priority	5	0	95.83	ODEQUS, NHSBT
	Number of consents (pmp)	High priority	5	1	96	Expert panel
	Number of families approached for deceased organ donation (pmp)	High priority	5	1	86.36	Expert panel
Donation	Mean number of organs donated and transplanted per deceased donor (by age group, co-morbidity, and donor type (DCD/DBD))	Very high priority	5	0	100	DSO
	DBD rate (pmp)	Very high priority	5	0	100	GODT
	Total living donation rate (by organ, pmp)	Very high priority	5	0	96	GODT
	DCD rate (pmp)	Very high priority	5	0	96	GODT
	Actual deceased organ donors (pmp)	Very high priority	5	0	95.83	AOTA
	Dedicated donor coordinator appointed in every public hospital with dedicated time	Very high priority	5	0	95.45	Expert panel
	DCD donors per number of deaths in hospital	Very high priority	5	0	95.45	Expert panel
	DCD donors per number of deaths	Very high priority	5	0	84	Expert panel
	Percentage of increased-risk donors	High priority	5	1	95.45	OPTN
	Percentage of consented donors with a recovered organ	High priority	5	1	92	CIHI
	Actual DCD donors from consented donors (pmp)	High priority	5	1	91.67	Expert panel
	Deceased donors per number of hospital deaths	High priority	5	1	90.91	Expert panel
	Number of kidney paired donations (pmp)	High priority	5	1	87.5	Expert panel
	People with registered intent to donate (pmp)	High priority	5	1	87.5	OPTN
	DBD donors per number of deaths	High priority	5	1	87.5	Expert panel
	Number of discarded kidneys (per number of kidneys procured)	High priority	5	1	86.36	OPTN (adapted)
	Corneal donation rate (pmp)	High priority	5	1	83.33	Expert panel
	Percentage of living kidney donors who develop end-stage kidney disease	High priority	5	1	83.33	OPTN
	Actual DBD donors from consented donors (pmp)	High priority	5	1	83.33	Expert panel
	Donor conversion index (donors per incidence of brain death)	High priority	4.5	1	87.5	Expert panel
	Percentage of audited deaths meeting referral criteria that were not referred	High priority	4.5	1	83.33	OPTN
	Donors per total number of intensive care beds	High priority	4.5	1	81.82	Expert panel
	Number of intensive care beds (per 100 000 population)	High priority	4.5	1	81.82	OECD
	Percentage of living kidney donors developing hypertension	High priority	4	1	90.91	Expert panel
	Percentage of living kidney donors developing proteinuria	High priority	4	1	86.36	Expert panel
	Percentage of individuals registered as a potential living donor who donated	High priority	4	1	83.33	OPTN
	Percentage of family donation conversations involving a donation specialist	High priority	4	1	81.82	AOTA
	Difference between the number of donors and the number of new patients added to waiting lists	High priority	4	1	80	Expert panel
Transplantation	Number of pre-emptive kidney transplantations (pmp)	Very high priority	5	0	100	Expert panel
	Number of transplants/number of patients on the waiting list (per organ)	Very high priority	5	0	100	Expert panel
	Number of liver-alone transplants (pmp)	Very high priority	5	0	86.36	OPTN
	Number of living donor kidney transplants (pmp)	Very high priority	5	0	100	OPTN
	Total transplantation rate (by organ, pmp)	Very high priority	5	0	100	GODT
	Thirty-day hospital readmission rate post-transplantation	High priority	5	1	100	Expert panel
	Average length of stay from admission to transplant, and transplant to discharge	High priority	5	1	86.36	OPTN
	Machine perfusion technology routinely used in transplantation	High priority	5	1	84	Expert panel
	Number of patients receiving more than one kidney transplant (by organ, pmp)	High priority	5	1	86.36	Expert panel
	Number of serious adverse events (per number of transplants)	High priority	5	1	83.33	DSO
	Number of serious adverse reactions (per number of transplants)	High priority	5	1	90.91	DSO
	Percentage of livers recovered for transplant but not transplanted	High priority	5	1	83.33	OPTN
	Total complication rate (by organ)	High priority	5	1	87.5	Expert panel
	Ratio of transplant patients undergoing reoperation in the first 15 days to total number of transplants	High priority	4	1	84	ODEQUS (adapted)
Follow-up	Total 10-year graft survival rate (by organ)	Very high priority	5	0	95.83	Expert panel
	Total 5-year graft survival rate (by organ)	Very high priority	5	0	100	ODEQUS (adapted)
	Total 1-year graft survival rate* (by organ)	Very high priority	5	0	98	OPTN
	Cumulative incidence of post-transplant dialysis (1 year)	High priority	5	1	91.67	OPTN
	Cumulative incidence of post-transplant dialysis (5 year)	High priority	5	1	86.96	OPTN
	Percentage of patients with appropriate screening and surveillance at 1, 5, and 10 years post-transplant	High priority	5	1	96	OPTN
	Percentage of recipients returning to work within 24 months post-transplant	High priority	5	1	81.82	Expert panel
	PROMs routinely used for assessment of transplantation outcomes	High priority	4.5	1	83.33	Expert panel
	Transplanted patients routinely referred for screening and intervention programmes to prevent cardiovascular risk factors	High priority	4	1	90.91	Expert panel
	Availability of counselling and psychosocial support for patients post-transplant	High priority	4	1	88	Expert panel

Indicator priority was based on a combination of median ratings, interquartile ranges, and the percentage of experts rating an indicator four or five. *Not included in original ranking; represents consolidated indicator based on the ranked indicators ‘total 1-year graft survival after pancreas transplantation’ and ‘total 1-year graft survival after kidney transplantation’. IT, information technology; NTO, national transplant organization; GODT, Global Observatory on Organ Donation and Transplantation; SES, socioeconomic status; pmp, per million population; ERA-EDTA, European Renal Association-European Dialysis and Transplantation Association; OPTN, Organ Procurement and Transplantation Network; DSO, Deutsche Stiftung Organtransplantation; AOTA, Australian Organ and Tissue Authority; ODEQUS, Organ Donation European Quality System; NHSBT, National Health Service Blood and Transplant; DCD, donation after circulatory death; DBD, donation after brain death; CIHI, Canadian Institute for Health Information; OECD, Organisation for Economic Co-operation and Development; PROMs, patient-reported outcome measures.

### Indicator prioritization

Based on expert ratings, the 84 indicators were stratified by priority level. A total of 25 indicators were categorized as very high priority (median rating = 5 and interquartile range = 0), representing the most strongly endorsed metrics across all domains. These very high priority indicators are presented separately in *Table 2* to facilitate rapid scorecard development for policymakers seeking a concise assessment tool.

**Table 2:** Indicators with very high priority

Category	Indicator
Monitoring and reporting	Patient satisfaction with transplant care recorded and used in transplant system performance assessment
	National IT platform dedicated to organ donation and transplantation in place
Waiting lists	Median days between being added to the waiting list and receiving transplant, for patients who received a transplant
	Median time awaiting first transplant (by organ)
	Total number of patients on waiting lists for organ transplantation per year (by organ, pmp)
	Total number of waiting list registrations per year (by organ, pmp)
	Waiting list is managed according to standardized criteria and international guidelines
Consent	Consent to organ donation, relative to number of people asked
	Family refusal rate
Donation	Mean number of organs donated and transplanted per deceased donor (by age group, co-morbidity, and donor type (DCD/DBD))
	DBD rate (pmp)
	Total living donation rate (by organ, pmp)
	DCD rate (pmp)
	Actual deceased organ donors (pmp)
	Dedicated donor coordinator appointed in every public hospital with dedicated time
	DCD donors per number of deaths in hospital
	DCD donors per number of deaths
Transplantation	Number of pre-emptive kidney transplantations (pmp)
	Number of transplants/number of patients on the waiting list (per organ)
	Number of liver-alone transplants (pmp)
	Number of living donor kidney transplants (pmp)
	Total transplantation rate (by organ, pmp)
Follow-up	Total 10-year graft survival rate (by organ)
	Total 5-year graft survival rate (by organ)
	Total 1-year graft survival rate (by organ)

IT, information technology; pmp, per million population; DCD, donation after circulatory death; DBD, donation after brain death.

**Fig. 2:**
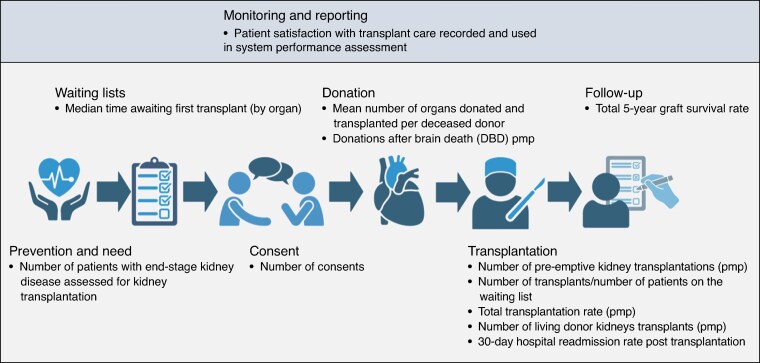
Highest-rated indicator(s) per domain

*Figure 2* illustrates the highest-rated indicator(s) within each of the seven domains. Indicators that did not achieve consensus for inclusion or were excluded for other reasons are presented in *Table S1*.

## Discussion

This study presents a set of indicators for transplant system performance measurement that incorporates established metrics alongside indicators reflecting prevention, patient-centred care, and equitable care. These performance indicators provide researchers and policymakers with a multifaceted overview of a transplant system’s strengths and weaknesses compared with systems in other countries. Indicators should be used together and in combination with qualitative assessment of transplant systems. This combined approach aims to identify potential areas for improvement in a nuanced way, thereby facilitating policy reforms that can enhance both system efficiency and patient outcomes across diverse healthcare contexts.

A set of 84 indicators for assessing transplant system performance was developed based on the consensus process. The indicators are distributed across seven domains covering the complete organ donation and transplantation pathway. The process also identified 25 very high priority indicators that represent the most strongly endorsed metrics across all domains.

The majority of indicators in the final list are drawn from existing resources in the field of organ donation and transplantation. Donation and transplantation rates, for example, serve as crucial components in any scorecard assessing transplant system performance, encompassing various donation types and organ systems. Consequently, they are collected and reported at the European level and are already used for policy analysis^9,13,38^. These rates are typically expressed per million population, allowing comparison across countries, and are accessible through the GODT^9^.

However, demographic differences between countries influence the potential for organ donation^39^. To account for these differences, international comparisons can incorporate mortality data from causes of death relevant to organ donation^27^. This enables donation rates to be understood in relation to the ‘potential’ for organ donation in the form of a ‘donor conversion index’, which has been included in the list of indicators (see *Table 1*). This could represent an advance compared with previous attempts to capture transplant system performance^9,27^, but would rely on timely and accurate reporting of mortality data.

Other indicators in the realm of prevention and need, focusing on kidney disease, are already systematically collected via the European Renal Registry^40^, but are not currently used in international transplant system comparisons. While the prevention of organ failure is of significant importance within any healthcare system^41^, there may be hesitance to include preventive indicators in a national transplant system performance scorecard due to challenges of attribution. These indicators likely reflect the performance of public health authorities and primary care, as well as socioeconomic factors, rather than the performance of transplant institutions. Correspondingly, it is not surprising that the indicators with consensus in this realm focus on tertiary rather than primary prevention of organ failure.

While donation and transplantation rates, waiting list parameters, and consent rates are already systematically collected and reported at a European level^9,38^, other indicators in the final list, particularly those addressing patient-centred and equitable care, are less well established in the field of transplantation.

Patient-centred care requires that quality metrics incorporate considerations important to both clinicians and patients^42,43^. This is reflected in the set of indicators. For example, 15-day reoperation rates effectively capture short-term complications and reflect the quality of surgical care^44,45^. Taking a longer-term perspective, the indicator set also incorporates outcomes such as 10-year graft survival. The preliminary expert consultations indicated that focusing on short intervals for graft survival might bias attention toward immediate postoperative management and excessive immunosuppression, whereas long-term outcomes are more relevant from a patient perspective.

The indicator set further incorporates patient-reported outcomes, including chronic pain and return-to-work status, which assess dimensions of system performance not captured by clinical or surgical endpoints alone^46,47^. This patient-centred perspective is reflected at both the system level (through monitoring and reporting requirements) and the individual level (for example follow-up indicators measuring the percentage of patients returning to work within 24 months post-transplantation).

Patient-reported outcome measures (PROMs) offer a structured way to integrate patient perspectives into routine practice^48,49^ and have demonstrated value in transplantation contexts^50–53^. Reflecting this importance, the expert panel prioritized an indicator assessing their routine use. However, current limitations exist in the transplantation field: transplant-specific PROMs are lacking, few address all transplanted organs comprehensively, and none are systematically collected at the European level^53^. A promising development in this area is a recently validated transplant-specific questionnaire that could potentially serve as a standardized data collection tool across European transplant systems in the future^54^.

Beyond outcomes, patient-reported experience measures (PREMs) represent another important dimension for future scorecard development^3,55^. While some existing surveys incorporate patient experience elements within chronic kidney disease assessments^56,57^, transplant-specific PREMs are not yet collected on an international scale^58,59^. The ‘Being Taken Seriously Questionnaire’ offers potential in this area, as it has been validated in high-tech hospital settings, is available in multiple languages, and can be applied across various organ transplantation contexts^60^.

In addition to patient-centred metrics, the indicator set includes metrics to assess equity in transplantation access. These specifically examine whether NTOs report data stratified by socioeconomic status (SES) and address equity in their reporting mechanisms. These indicators reflect the principle that healthcare access should be based on clinical need rather than SES, sex, race/ethnicity, or location^55,61,62^.

Research has established clear disparities in transplantation, with studies showing reduced kidney transplant listing rates among individuals with lower SES in the UK^51^ and worse access for women compared with men in the USA^52^. Despite recognition of these inequities by European and American transplantation societies^63,64^, many European NTOs fail to disaggregate their data by critical sociodemographic factors in national reports^65^ and no transplant-specific surveys measure self-reported unmet need. This creates a significant gap in understanding the full scope of disparities across different patient populations and regions.

Accordingly, the indicator set includes metrics to assess performance data disaggregated by relevant sociodemographic characteristics and whether equity considerations feature in routine reporting. NTOs should implement standardized measurement frameworks to quantify these disparities as an essential step toward addressing inequitable access patterns and establishing accountability mechanisms within transplant systems. Future indicator development should expand to capture geographical variations in access to evaluation, waitlisting, and transplantation, providing a more comprehensive understanding of equity challenges across the transplantation care continuum.

This set of validated performance indicators can be used by researchers and policymakers in multiple ways. Policymakers can build a scorecard based on these validated performance indicators using various approaches, for example: selecting indicators with the highest consensus by domain (*Fig. 2*); focusing on indicators with very high priority (*Table 2*); or selecting a combination of indicators according to their specific needs and priorities. Recognizing that data availability varies across transplant systems, initial implementation efforts might begin with a minimum set of indicators based on data already routinely collected, with the indicator set expanded over time as monitoring capacity develops. In this way, the full indicator set presented here can inform future efforts to strengthen data collection and use. The scorecard can then be used to assess the current status of transplant systems, identify areas for further investigation and improvement, and monitor the effects of reform efforts over time. A scorecard based on the presented indicators could also enable rapid comparison with other transplant systems worldwide to identify strengths, weaknesses, and international best practices. The list of indicators, covering multiple elements of a successful transplant system, provides the basis for more nuanced comparison than donation or transplantation rates alone.

When conducting international comparisons, policymakers should interpret these indicators as diagnostic tools rather than as a basis for ranking or performance league tables. Indicators such as waiting list parameters require careful interpretation; for example, the number of patients on a waiting list reflects the inclusivity of listing practices as much as transplantation rates and a high number does not necessarily imply poor system performance. Consequently, individual metrics should be analysed together and triangulated with qualitative evidence, for instance audits of listing protocols, prioritization criteria, and the ethical governance of allocation processes. Employed within such a mixed-methods evaluative strategy, the framework can inform policy development while mitigating the risk of reductive conclusions about complex transplant systems.

This study focuses on European transplant systems, which is reflected in the composition of the expert panel and in the source documents, including several reports developed through initiatives of European institutions^23,66,67^. Because this review covered a range of health systems, including countries with relatively limited financial capacity^15,16^, the findings are broadly applicable across European settings and may also inform approaches in non-European high-resource environments. However, due to the emphasis on best-practice examples and the composition of the expert panel, the resulting indicator list tends to reflect well-developed transplant systems. This is illustrated by indicators related to procedures such as donation after circulatory death (DCD) or machine perfusion, which require adequate technical and workforce resources. It is also reflected in indicators that depend on robust data monitoring capacities, such as 10-year graft survival, patient-reported outcomes, and hospital readmission rates. These indicators may be challenging to implement in settings without systematic data collection or where resource constraints limit high-cost interventions. Consequently, some indicators may represent long-term aspirational goals rather than immediately applicable metrics in all European contexts.

Nonetheless, evidence from the Portuguese transplant system illustrates that implementing a transplant-specific national information technology (IT) infrastructure is possible despite resource constraints^16^. Similarly, PROMs can be adapted and successfully implemented even in low-resource settings^68,69^, provided that surveys are appropriately translated and tailored to local contexts^69–71^. Thus, even countries with limited resources or less sophisticated transplant infrastructures may benefit from the use of these indicators. The research team is currently collaborating with the Greek organ donation and transplantation authorities to explore how these indicators can be applied in an evolving system that has historically faced resource and infrastructural challenges^14^. One possible approach is to combine routine indicators, such as donation and transplantation rates, with a small number of novel indicators, for example those assessing the implementation of basic patient-reported outcomes. This approach would allow for more nuanced assessment than relying solely on donation rates while remaining feasible across different settings. Finally, beyond system resources, applicability may also vary according to cultural context. For instance, DCD is not universally pursued in Europe and may not be applicable due to reasons beyond system sophistication. Factors such as cultural acceptability or ethical considerations might influence the adoption of this form of donation^72^.

The study’s strength lies in its multidimensional approach and validation by a diverse expert group, inclusive of patient representation. Its combination of open preliminary expert discussions with a modified Delphi approach promotes high internal validity, as it allows for a rigorous ranking process while maintaining flexibility and face validity. However, certain limitations should be noted. First, the study’s reliance on reviewing existing resources and a subset of NTOs represents a systematic yet not exhaustive method for identifying potential indicators. Furthermore, due to the emphasis on best-practice examples and the composition of the expert panel, some indicators may represent long-term aspirational goals rather than immediately applicable metrics in all European contexts, particularly in settings with resource or infrastructural constraints.

Data availability and comparability emerge as significant concerns within the indicator set. Indicators like donation and transplantation rates are readily accessible from public European databases, comparable across countries, and regularly updated^9,38^. Others, such as waiting list statistics, can be accessed through the ‘Newsletter Transplant’ series^38^, but might be challenging to compare across different systems due to diverse definitions and inclusion criteria^73^. This is particularly true for indicators based on cause-specific mortality data, which are known to vary significantly both between and within countries^74^. Other indicators, such as the number of patients reporting chronic pain or total 10-year graft survival, pose challenges in data availability. Nonetheless, it is hoped that this study will prompt and encourage the collection and standardization of indicators that are not currently easily accessible^75^.

## Supplementary Material

znaf293_Supplementary_Data

## Data Availability

The data supporting the findings of this study, including the full list of evaluated indicators and anonymized expert ratings, are available from the corresponding author upon request.
