# Supplementary material for: Performance indicators for organ donation and transplantation programmes in Europe: modified Delphi consensus study
Source: Br J Surg. 2026 Jan 22;113(1):znaf293. doi: 10.1093/bjs/znaf293 (PMC12825608; doi:10.1093/bjs/znaf293)
Supplement: znaf293_Supplementary_Data [file znaf293_supplementary_data.docx]

**Performance Indicators for Organ Donation and Transplantation Programmes in Europe: a Modified Delphi Consensus Study**

**Authors:** Simon Streit¹, George Wharton², Jasmine Mah³, Robin von Kessel², Apostolos Prionas⁴, Charlotte Johnston-Webber², John Boletis⁵, Beatriz Domínguez-Gil⁶, Anna Forsberg⁷, Ana França⁸, Dale Gardiner⁹, Patrick Jeurissen¹⁰, Irene Papanicolas², Oliver Pearcey¹¹, Allan Rasmussen¹², Jacopo Romagnoli¹³, Elias Mossialos², and Vassilios Papalois⁴

**Affiliations:**

*¹ Charité Universitätsmedizin, Institut für Neuropathologie, Berlin, Germany
² London School of Economics, Department of Health Policy, London, United Kingdom
³ Dalhousie University, Division of Geriatric Medicine, Halifax, Canada
⁴ Imperial College, Department of Surgery, London, United Kingdom
⁵ Laiko Hospital, Transplantation Unit, Athens, Greece
⁶ Organización Nacional de Trasplantes, Madrid, Spain
⁷ Lund University, Department of Health Sciences, Lund, Sweden
⁸ Instituto Português do Sangue e da Transplantação, Lisbon, Portugal
⁹ NHS Blood and Transplant, Bristol, United Kingdom
¹⁰ IQ Healthcare Scientific Institute for Quality of Healthcare, Radboud Institute for Health Sciences, Nijmegen, Netherlands
¹¹ West London Kidney Patients' Association, London, United Kingdom
¹² Rigshospitalet, University of Copenhagen, Department of Surgical Gastroenterology and Transplantation, Copenhagen, Denmark
¹³ Fondazione Policlinico Universitario A. Gemelli IRCCS, Dipartimento di Scienze Mediche e Chirurgiche, U.O.S. Trapianti di Rene, Roma, Italy*

**Corresponding author:** George Wharton, Department of Health Policy, London School of Economics and Political Science, London, WC2A 2AE, United Kingdom, [g.a.wharton@lse.ac.uk](mailto:g.a.wharton@lse.ac.uk), ORCID: 0000-0001-6544-3636

**Supplementary Materials - Index**

| **Supplementary Figures and Tables** |  |
| --- | --- |
| Supplementary Table 1. Indicators excluded from final set of indicators | *page 2* |
| Supplementary Table 2: STROBE Checklist - Cross-sectional Study | *page 5* |

**Supplementary Figures and Tables**

**Supplementary Table 1. Indicators excluded from final set of indicators**

This includes indicators where no expert consensus could be reached, indicators that were excluded based on expert ratings and indicators that were consolidated or deleted by the research team as part of the systematic ranking process.

| **Indicator** | **Median** | **IQR** | **% 4/5** | **Priority** | **Reason for Exclusion** |
| --- | --- | --- | --- | --- | --- |
| 1-year graft survival rate after pancreas transplantation | 5 | 0 | 100 | Very high | Consolidated |
| Number of heart transplants (per million population) | 5 | 0 | 100 | Very high | Consolidated |
| Number of liver transplants (per million population) | 5 | 0 | 100 | Very high | Consolidated |
| Number of patients in kidney waiting list (per million population) | 5 | 0 | 100 | Very high | Consolidated |
| 1-year graft survival rate after kidney transplantation | 5 | 0 | 96 | Very high | Consolidated |
| 5-year graft survival rate after kidney transplantation | 5 | 0 | 96 | Very high | Consolidated |
| 5-year graft survival rate after pancreas transplantation | 5 | 0 | 96 | Very high | Consolidated |
| Number of kidney transplants (per million population) | 5 | 0 | 96 | Very high | Consolidated |
| Number of lung transplants (per million population) | 5 | 0 | 96 | Very high | Consolidated |
| Number of pancreas transplants (per million population) | 5 | 0 | 96 | Very high | Consolidated |
| Number of patients in heart waiting list (per million population) | 5 | 0 | 95 | Very high | Consolidated |
| Number of living donor liver transplants (per million population) | 5 | 1 | 92 | High | Consolidated |
| Number of simultaneous pancreas-kidney transplants (per million population) | 5 | 1 | 92 | High | Consolidated |
| Number of new registrations on waiting lists per year (by organ, per million population) | 5 | 1 | 88 | High | Consolidated |
| Number of pancreas alone transplants (per million population) | 5 | 1 | 88 | High | Consolidated |
| Number of patients on combined kidney and pancreas waiting list (per million population) | 5 | 1 | 88 | High | Consolidated |
| Number of patients on pancreas alone transplant waiting list (per million population) | 5 | 1 | 88 | High | Consolidated |
| Number of small bowel transplants (per million population) | 5 | 1 | 88 | High | Consolidated |
| Number of pancreas after kidney transplants (per million population) | 4.5 | 1 | 83 | High | Consolidated |
| Viral infection or reactivation rate (per number of transplants) | 4 | 1 | 77 | Moderate | No Consensus |
| Average time between first and second transplant (by organ) | 4.5 | 1 | 77 | Moderate | No Consensus |
| Family overturn rate | 5 | 1 | 77 | Moderate | No Consensus |
| Number of transplants per expenditure on healthcare system | 5 | 1 | 77 | Moderate | No Consensus |
| Percentage of transplantation processes stopped | 4.5 | 1 | 77 | Moderate | No Consensus |
| Average time between referral and approach | 4 | 1 | 77 | Moderate | No Consensus |
| Cost per transplant by type of transplant | 5 | 2 | 73 | Moderate | No Consensus |
| Incidence of patients accepted for renal replacement therapy (unadjusted) | 4 | 2 | 73 | Moderate | No Consensus |
| Number of simultaneous liver-kidney transplants (per million population) | 4 | 2 | 73 | Moderate | No Consensus |
| Percentage of kidney transplant recipients with new-onset diabetes | 4 | 2 | 73 | Moderate | No Consensus |
| Percentage of living donors not meeting clinical guidelines at follow up | 4 | 2 | 73 | Moderate | No Consensus |
| Number of organs transferred to other countries not as part of organ exchange programmes | 4.5 | 2 | 68 | Moderate | No Consensus |
| Cost per kidney transplant, ratio to cost of dialysis per annum | 5 | 2 | 68 | Moderate | No Consensus |
| Number of organ donors who become corneal donors | 5 | 2 | 68 | Moderate | No Consensus |
| Percentage of people who indicated mistrust in the transplant system (per million population) | 4 | 1 | 68 | Moderate | No Consensus |
| Number of patients with left ventricular assistance device | 4 | 2 | 64 | Moderate | No Consensus |
| Prevalence of diabetes (per million population) | 4 | 3 | 64 | Moderate | No Consensus |
| Transplant system budget in relation to general health system expenditure | 5 | 2 | 64 | Moderate | No Consensus |
| Total number of transplants since the establishment of the national transplant organisation | 5 | 3 | 59 | Moderate | No Consensus |
| Number of donation-related contacts with the National Transplant Organisation (per million population) | 4 | 1 | 59 | Moderate | No Consensus |
| Percentage of consultations where the patient was accepted for transplant | 3.5 | 3 | 50 | Moderate | No Consensus |
| Ratio of Heart transplants to Lung transplants | 3.5 | 1 | 50 | Moderate | No Consensus |
| Daily exposure to tobacco smoke indoors | 2 | 1 | 12 | Low | Expert Consensus |
| Is fear of graft rejection recorded in post transplant follow up as part of patient experience surveys | 3 | 1 | 48 | Low | Expert Consensus |
| Percentage of transplant patients with chronic pain | 3 | 3 | 48 | Low | Expert Consensus |
| Number of hospital admissions for diabetes mellitus (per million population) | 3 | 2 | 46 | Low | Expert Consensus |
| Percentage of adult population that is overweight (BMI>25) | 3 | 3 | 42 | Low | Expert Consensus |
| Percentage of patients screened for high blood sugar and received a medical intervention (percentage of the population screened and treated) | 3 | 2 | 39 | Low | Expert Consensus |
| National anti-tobacco policy in place | 2 | 2 | 38 | Low | Expert Consensus |
| Percentage of patients screened for high blood pressure by a health professional within the last year | 3 | 3 | 38 | Low | Expert Consensus |
| Percentage of patients that were screened for high cholesterol and received a medical intervention (percentage of the population screened and treated). | 3 | 2 | 38 | Low | Expert Consensus |
| Percentage of people reporting doing health-enhancing physical activity at least once a week | 2.5 | 2 | 38 | Low | Expert Consensus |
| Frequency of alcohol consumption | 3 | 2 | 36 | Low | Expert Consensus |
| Percentage of patients that were screened for high blood pressure and received a medical intervention (percentage of the population screened and treated). | 3 | 2 | 36 | Low | Expert Consensus |
| Percentage of patients screened for high blood sugar by a health professional within the last year | 3 | 2 | 33 | Low | Expert Consensus |
| Percentage of patients screened for high cholesterol by a health professional within the last year | 3 | 2 | 33 | Low | Expert Consensus |
| Proportion of people reporting doing non-work-related physical activities at least once a week | 3 | 2 | 33 | Low | Expert Consensus |
| Proportion of people reporting hazardous alcohol consumption | 2.5 | 2 | 33 | Low | Expert Consensus |
| Proportion of daily smokers of tobacco products | 2 | 2 | 32 | Low | Expert Consensus |
| Number of hospital admissions for diabetic ketoacidocis (per million population) | 2 | 2 | 32 | Low | Expert Consensus |
| Proportion of current smokers of tobacco products | 2 | 2 | 29 | Low | Expert Consensus |
| Time spent on health-enhancing aerobic physical activity | 2 | 3 | 28 | Low | Expert Consensus |
| Number of hospital admissions for hyperglycaemic emergency (per million population) | 2 | 2 | 27 | Low | Expert Consensus |
| Number of patients with diabetes with lower-extremity amputation (per million population) | 3 | 1 | 25 | Low | Expert Consensus |
| Duration of daily tobacco smoking | 2 | 2 | 24 | Low | Expert Consensus |
| Number of hospital admissions for hypertension (per million population) | 2 | 1 | 24 | Low | Expert Consensus |
| Frequency of heavy episodic drinking | 2 | 2 | 21 | Low | Expert Consensus |
| Number of portions of fruits and vegetables consumed per day | 2 | 2 | 21 | Low | Expert Consensus |
| Use of electronic cigarettes or similar electronic devices | 2 | 1 | 17 | Low | Expert Consensus |
| Frequency of drinking sugar-sweetened soft drinks | 2 | 1 | 13 | Low | Expert Consensus |
| Frequency of fruit and vegetables consumption | 2 | 2 | 8 | Low | Expert Consensus |
| Frequency of drinking pure fruit or vegetable juice | 2 | 1 | 4 | Low | Expert Consensus |
| Total number of new patients added to waiting lists per year (by organ, per million population) | 5 | 1 | 96 | High | Deleted (duplicate) |
| Number of consents per million population, change over 10 years | 5 | 1 | 84 | High | Deleted (redundant) |
| Family refusal rate, change over 10 years | 5 | 1 | 83 | High | Deleted (redundant) |
| Number of patients in liver waiting list for at least one day (per million population) | 5 | 1 | 79 | Moderate | Deleted (redundant) |
| Annual change in deceased donors | 5 | 1 | 79 | Moderate | Deleted (redundant) |
| Number of patients in lung waiting list for at least one day (per million population) | 5 | 1 | 76 | Moderate | Deleted (redundant) |
| Number of organs exported to other countries | 4 | 1 | 76 | Moderate | Deleted (face validity) |
| Percentage of successful organ donation after contact with the NTO | 5 | 1 | 76 | Moderate | Deleted (feasibility) |
| Change in the number of transplantations from last year (per million population) | 4 | 1 | 75 | Moderate | Deleted (redundant) |
| Number of patients on the intestine transplant waiting list for at least one day (per million population) | 4 | 2 | 71 | Moderate | Deleted (redundant) |
| Ethnicity and SES of recipients match age and SES of patients in need for organ transplantation | 4 | 2 | 67 | Moderate | Deleted (feasibility) |
| Number of transplants per expenditure on transplant system | 4 | 2 | 63 | Moderate | Deleted (feasibility) |
| Number of organs in surplus exported to other countries | 4 | 2 | 63 | Moderate | Deleted (edited) |
| Time to return on investment for kidney transplantation compared to dialysis | 4 | 2 | 54 | Moderate | Deleted (feasibility) |

**Supplementary Table 2: STROBE Checklist - Cross-sectional Study**

| Item No | Recommendation | Page No/Section |
| --- | --- | --- |
| Title and abstract |  |  |
| 1 | (a) Indicate the study's design with a commonly used term in the title or the abstract | Abstract: "modified Delphi consensus process" |
|  | (b) Provide in the abstract an informative and balanced summary of what was done and what was found | Abstract: structured format with Background, Method, Results, Conclusion |
| Introduction |  |  |
| 2 | Explain the scientific background and rationale for the investigation being reported | Introduction: paragraphs 1-3 |
| 3 | State specific objectives, including any prespecified hypotheses | Introduction: final paragraph |
| Methods |  |  |
| 4 | Present key elements of study design early in the paper | Methods: "Study Design" section |
| 5 | Describe the setting, locations, and relevant dates, including periods of recruitment, exposure, follow-up, and data collection | Methods: "conducted between June and November 2024 using the Welphi platform, a specialised web application" |
| 6 | (a) Give the eligibility criteria, and the sources and methods of selection of participants | Methods: "Modified Delphi Consensus Process" - expert selection criteria |
| Variables |  |  |
| 7 | Clearly define all outcomes, exposures, predictors, potential confounders, and effect modifiers. Give diagnostic criteria, if applicable | Methods: "5-point Likert scale" and consensus definitions |
| Data sources/measurement |  |  |
| 8 | For each variable of interest, give sources of data and details of methods of assessment (measurement). Describe comparability of assessment methods if there is more than one group | Methods: "Welphi platform" and rating methodology |
| Bias |  |  |
| 9 | Describe any efforts to address potential sources of bias | Methods: "indicators were presented in randomised order" |
| Study size |  |  |
| 10 | Explain how the study size was arrived at | Methods: "30 international experts" - purposive sampling |
| Quantitative variables |  |  |
| 11 | Explain how quantitative variables were handled in the analyses. If applicable, describe which groupings were chosen and why | Methods: "consensus for inclusion as ≥80%", priority categories |
| Statistical methods |  |  |
| 12 | (a) Describe all statistical methods, including those used to control for confounding | Methods: "Statistical analysis was performed in R" |
|  | (b) Describe any methods used to examine subgroups and interactions | N/A |
|  | (c) Explain how missing data were addressed | Methods: "including both complete and incomplete responses" |
|  | (d) If applicable, describe analytical methods taking account of sampling strategy | N/A |
|  | (e) Describe any sensitivity analyses | Methods: Second round methodology for indicators with moderate approval |
| Results |  |  |
| Participants |  |  |
| 13 | (a) Report numbers of individuals at each stage of study—eg numbers potentially eligible, examined for eligibility, confirmed eligible, included in the study, completing follow-up, and analysed | Results: "30 experts agreed to participate...24 experts (80%) completed round 1 and 22 (73%) completed round 2" |
|  | (b) Give reasons for non-participation at each stage | N/A - not reported |
|  | (c) Consider use of a flow diagram | Figure 1: Consensus Process flowchart |
| Descriptive data |  |  |
| 14 | (a) Give characteristics of study participants (eg demographic, clinical, social) and information on exposures and potential confounders | Results: participant professions and countries |
|  | (b) Indicate number of participants with missing data for each variable of interest | Results: response rates by round |
| Outcome data |  |  |
| 15 | Report numbers of outcome events or summary measures | Results: "103 achieved consensus", Table 1 |
| Main results |  |  |
| 16 | (a) Give unadjusted estimates and, if applicable, confounder-adjusted estimates and their precision (eg, 95% confidence interval). Make clear which confounders were adjusted for and why they were included | Results: median ratings, IQR, consensus percentages in Table 1 |
|  | (b) Report category boundaries when continuous variables were categorized | Methods: priority categories defined |
|  | (c) If relevant, consider translating estimates of relative risk into absolute risk for a meaningful time period | N/A |
| Other analyses |  |  |
| 17 | Report other analyses done—eg analyses of subgroups and interactions, and sensitivity analyses | Supplementary Table 1: excluded indicators |
| Discussion |  |  |
| Key results |  |  |
| 18 | Summarise key results with reference to study objectives | Discussion: paragraph 1 |
| Limitations |  |  |
| 19 | Discuss limitations of the study, taking into account sources of potential bias or imprecision. Discuss both direction and magnitude of any potential bias | Discussion: "Strengths and Limitations" section |
| Interpretation |  |  |
| 20 | Give a cautious overall interpretation of results considering objectives, limitations, multiplicity of analyses, results from similar studies, and other relevant evidence | Throughout Discussion section |
| Generalisability |  |  |
| 21 | Discuss the generalisability (external validity) of the study results | Discussion: limitations regarding geographic and cultural contexts |
| Other information |  |  |
| Funding |  |  |
| 22 | Give the source of funding and the role of the funders for the present study and, if applicable, for the original study on which the present article is based | Title page: "No funding" |

*N/A = Not applicable to this study design

**Note:** This checklist is based on the STROBE Statement for cross-sectional studies. For more information, visit [www.strobe-statement.org](http://www.strobe-statement.org/)
